# Integrating Occupational Health and Safety and Health Promotion: A Qualitative Study in Australia

**DOI:** 10.1002/hpja.70173

**Published:** 2026-03-16

**Authors:** Yanming Lu, Nektarios Karanikas, Julie‐Anne Carroll

**Affiliations:** ^1^ School of Public Health and Social Work, Faculty of Health Queensland University of Technology Kelvin Grove Queensland Australia

**Keywords:** employer, health promotion, integration, occupational health and safety, professional

## Abstract

**Issue Addressed:**

Growing evidence supports integrating occupational health and safety (OHS) and workplace health promotion (WHP) to enhance worker health, safety and wellbeing. This study aimed to explore in Australia: (1) the reasons for implementing OHS and WHP separately or concurrently/jointly; and (2) potential preferences and implementation contexts of integrated OHS‐WHP approaches.

**Methods:**

This qualitative study involved focus groups (*n* = 3) and individual interviews (*n* = 40) with 47 OHS professionals, health promotion (HP) professionals, occupational health professionals and employers in Australia. Data were collected between October 2024 and March 2025. A thematic inductive analysis was employed.

**Results:**

Several important insights include: (1) knowledge and attitudes of professionals towards OHS or WHP, particularly regarding OHS professionals' limited understanding of WHP and HP professionals' difficulties with OHS terminology; (2) reasons for inadequate integration implementation, including scarce WHP implementation, invisible WHP benefits, poor WHP evaluation, differing views of OHS professionals about WHP, debate about worker health and WHP quality and implementation issues; (3) trends and examples of integration, often motivated by factors like COVID‐19 and occurring more successfully in large organisations, though often hindered by departmental siloing; and (4) roles, attitudes and knowledge of employers, with varying support for integration across management levels and organisational sizes.

**Conclusions:**

Limited implementation of integrated OHS‐WHP approaches in Australia stems from lack of policy emphases, professional knowledge gaps, and WHP implementation and evaluation concerns.

**So What?:**

Urgent educational opportunities for employers and OHS professionals are needed to foster a holistic consideration of worker health and wellbeing, encouraging meaningful collaboration with health professionals and applying systems thinking to contemporary WHP.

## 1 Introduction

In the context of enhancing worker health, safety and wellbeing, growing evidence supports the integration of occupational health and safety (OHS) and workplace health promotion (WHP) interventions [1]. Each contains its relatively distinct intervention scope, despite some potential overlaps. OHS predominantly comprises the strategies for reducing workplace ergonomic, psychosocial, and material risks (e.g., physical, chemical, biological) to manage work‐related diseases and injuries. In contrast, WHP aims for individual‐based health education and health behaviour changes (e.g., health literacy, access to health services) [2, 3].

The expected benefits of the integrated OHS‐WHP approaches broadly consist of increased worker participation, effective disease prevention and treatment, reduction of occupational injuries and disabilities, lower healthcare and social costs, and improved worker productivity and morale [4, 5, 6, 7]. The preliminary results show that integrated approaches could be more effective than OHS or WHP being implemented separately [1, 5, 7]. However, integration effectiveness depends on a range of factors, such as appropriate use of resources, leadership support, and intervention tailoring [1, 5, 7], necessitating a good intervention planning process.

OHS systems in most countries are well established and legally enforced, including comprehensive and effective risk reporting and control systems. Internationally, employers are generally considered legally responsible to ensure managing the OHS risks as far as is reasonably practicable, with workers also required to attend to their own health and safety, including compliance with reasonable instructions by their workplaces [8, 9]. WHP is, however, considered a relatively emerging service area, based principally on the notion of voluntary and encouragement‐based implementation and participation [10].

For instance, in the United States, WHP has been covered by most public and private health insurance schemes, with good outcomes (e.g., care coordination for chronic disease management and medication compliance, comprehensive health and wellness initiatives for hospital discharge) [11]. Yet, in Australia, WHP‐related systematic evidence is poorly documented owing to its immature development and inadequate policy support [10]. Moreover, given that specific OHS challenges, failures, and enforcement measures in Australia remain largely confidential [12], the public available OHS‐related information is scarce, concurrently rendering the evidence of integrated approaches scant. This warrants research to inform best evidence‐based opportunities, challenges, and practices of integrated approaches in Australia.

Furthermore, despite the growing research globally, the lack of consistent and clear definitions of integrated approaches within the literature renders the extant findings less transferrable in different workplace settings [13, 14, 15, 16]. Other evidence portrays such approaches by adopting abstract and philosophical language, without specific and tangible intervention content or scope [16]. These observations reveal that not only does evidence related to integrated approaches remain problematic, but the ambiguity of the scope of and relationship between OHS and WHP may also lead to confusion and poor planning, implementation, and evaluation at multiple levels.

However, the results from a recent nation‐wide cross‐sectional study conducted in Australia, which was informed by four types of needs assessment by Bradshaw (i.e., expert/normative: OHS professionals; felt: workers; expressed: administrative staff; comparative: similar contexts) [17], provide preliminary results highlighting several areas for intervention efforts at the worker level (i.e., felt needs) [18]. These key areas when planning OHS‐WHP integrated approaches include supporting frequent breaks (49%), providing training and education (33%), offering mental health support (19%), enhancing risk management (19%), providing ergonomic workstations (15%), organising recreational and physical activities (15%), and the use of Personal Protective Equipment (6%) [18].

These seven areas, while considered to some extent relatively broad, may offer useful insights into ‘how to start planning integrated approaches meaningfully’. Further, this cross‐sectional study observed that large‐sized organisations were more likely to implement integrated approaches and that there existed limited implementation (25%) of integrated approaches in Australian workplaces [19]. This research, however, has a few limitations. For example, the majority of the responses from open‐ended questions were described in a succinct manner and did not offer more detailed contextual information.

Also, the survey participants were solely focused on workers, without any other stakeholders' opinions (e.g., employers, health and safety professionals) to balance and complement the analysis. Nonetheless, to supplement and contextualise further the results above and advance the understanding of integrated approaches, it is imperative to collect in‐depth and detailed information from employers and OHS and health professionals, representing expert/normative and expressed needs and professional opinions and experiences from implementing and observing OHS and WHP services.

OHS professionals in general play a fundamental role in supporting employers to enhance worker health, safety and wellbeing; more specifically, OHS professionals are considered primary contact in the workplaces for any safety initiatives to meet enforced legal requirements. Besides, roles with a more health focus such as health promotion (HP) professionals and occupational health (OH) professionals (e.g., more clinical knowledge) usually collaborate with OHS professionals or work independently on particular health‐related campaigns [9]. Because of their roles and expertise, those experts are more likely to be actively involved in planning, implementing, and evaluating health and safety strategies in practice, thus being best situated to provide valuable first‐hand insights. Furthermore, the above professionals, as actual intervention implementers, may work in multiple workplaces (e.g., consultants) or have experience from difference enterprises [9]. Hence, they can offer comprehensive information (e.g., attitudes, behaviours, practices) related to planning, implementation, and evaluation across various contexts.

Overall, a collective understanding of the perspectives of workers, employers, and health and safety professionals is needed to build a relatively comprehensive picture of integrated approaches, thus significantly addressing the knowledge and practice deficiency in this field. Therefore, the current study aimed to explore integrated OHS‐WHP approaches in a sample of professionals and employers in Australia further to more holistically understand the following:

1. The reasons for implementing OHS and WHP separately or concurrently/jointly; and
2. Potential preferences and implementation contexts of integrated approaches.

## 2 Methods

### 2.1 Study Design and Sample

This study was conducted in Australia, and ethics approval was obtained from the university's Human Research Ethics Committee (approval number: 8350). We used a combination of interviews and focus groups to leverage the benefits from both data collection methods. Interviews are a well‐established method for understanding how interviewees attribute meanings to their experiences [20]. On the other hand, focus groups can also prove valuable sources of information because they allow participants to explain their opinions to each other and elicit deeper underlying meanings [21]. Hence, the combination of interviews and focus groups ensured both depth and breadth and allowed for capturing information emerging from both personal perspectives and experiences and interactions between subject matter experts.

The notion of the Knowledge to Action (KTA) Framework underpinned this study design [22]. The knowledge creation is an exploration and iterative process that analyses the existing knowledge (knowledge inquiry) to generate refined or new knowledge (knowledge synthesis) so as to develop knowledge tools or products [22]. The discussion topics used in the study are attached as Appendix A and served as a tool to encourage participants to think broadly and deeply about integrated approaches.

The development of the discussion topics was informed by the KTA Framework, a recent scoping literature review of integrated approaches and generic discussion guidance in the broader context of implementation science [1, 22, 23]. At the beginning of each session, the researchers provided the participants with tangible and clear definitions of integrated approaches to ensure a shared understanding of the topic [1]. Also, the researchers provided the results of the key intervention areas from the open‐ended questions of the recent cross‐sectional study (as discussed in Introduction) to ensure participants had a proper understanding of conceptions.

The recruitment strategy, informed by a purposive sampling approach, included word of mouth, existing professional network, social media posts (e.g., LinkedIn, Facebook), and advertisement through newsletters of professional associations, such as Australian Health Promotion Association, Public Health Association of Australia, Human Factors and Ergonomics Society of Australia, and Australian Institute of Health & Safety. Eligible participants were identified as OHS professionals, HP professionals, OH professionals, and employers currently working in Australia. Their professional identities were verified by the research team. Interested participants were provided with the options to attend either a focus group, an interview, or both. No reimbursement was provided for participation.

We conducted three online focus groups and 40 individual interviews in Australia between late October 2024 and early March 2025, with a total of 47 participants. All participants provided informed consent. Each focus group, lasting approximately 1 h, included four participants, with one or two moderators from the research team facilitating each session. Each interview lasted between 45–60 min, with two interviews conducted in person and the remaining online. Of all participants, only five attended both an interview and a focus group; the remaining attended either a focus group or an interview. To minimise the potential bias of focus group discussions changing individual perceptions and attitudes [21], these five participants first attended an interview before participating in a focus group.

Because of participant availability limitations, the composition of focus groups did not include a mix of OHS professionals, employers, OH professionals, and HP professionals that could serve to collect information from the interactions between stakeholders with varying roles and experiences. To redress this limitation, given that most participants held OHS consultancy‐related roles in engaging with various workplaces and professionals, the focus group moderator encouraged participants to think about other relevant professionals in any industry and context with whom they interacted and invited participants to describe corresponding experiences. In addition, some participants had multiple professional roles; for example, they identified as both OHS and HP professionals. The demographics of the study participants are listed in Table 1. More than half of the participants identified as OHS professionals, with the minority as HP, OH, and employers. Several OHS professionals had additional roles such as OH, HP, and employers.

**TABLE 1:** Demographics of focus group and interview participants.

Types of sessions (*n* = 47)^d^	Role
Focus Group 1 (*n* = 4)	Occupational Health and Safety & Occupational Health (*n* = 2)^a^
	Occupational Health and Safety & Health Promotion (*n* = 1)^a^
	Occupational Health and Safety (*n* = 1)
Focus Group 2 (*n* = 4)	Occupational Health and Safety (*n* = 3)^b^
	Occupational Health and Safety & Employer (*n* = 1)
Focus Group 3 (*n* = 4)	Occupational Health and Safety (*n* = 2)^c^
	Occupational Health and Safety & Occupational Health (*n* = 1)
	Occupational Health and Safety & Employer (*n* = 1)
Interview (*n* = 40)	Occupational Health and Safety & Occupational Health (*n* = 1)
	Occupational Health and Safety (*n* = 18)
	Health Promotion (*n* = 8)
	Employer (*n* = 8)
	Occupational Health (*n* = 5)

^a^ In focus group 1, two Occupational Health and Safety & Occupational Health professionals and one Occupational Health and Safety & Health Promotion professional attended both focus groups and interviews.

^b^ In focus group 2, one Occupational Health and Safety professional attended both a focus group and an interview.

^c^ In focus group 3, one Occupational Health and Safety professional attended both a focus group and an interview.

^d^ *Note:* Five participants attended both focus groups and interviews.

### 2.2 Data Collection and Analysis

The focus groups and interviews were audio recorded and transcribed verbatim through the university‐approved online transcription service that meets ethical research standards. Transcripts were then imported into NVivo Version 15 for systematically analysing and presenting qualitative data [24]. We employed a reflective thematic inductive (i.e., data‐driven) analysis approach informed by the steps presented by Braun and Clarke [25]. This afforded the necessary flexibility in data analysis [26], particularly suitable for this study design. The research team involved researchers with extensive experience and expertise in occupational health and safety and health promotion and in analysing qualitative data.

The transcripts were initially reviewed by the research team and the audio recordings were frequently listened to ensure familiarity with the data. Each transcript was then open‐coded independently by two members of the research team who employed an inductive approach to identify initial concepts, patterns, and similarities. Given the latest insights into good practices of reflective thematic analysis by Braun and Clarke [27], we employed several approaches to best enhance methodological congruence. First, key implications from the data were systematically examined and coded to capture original meanings and ensure they closely linked to relevant interpretations. Second, we considered all codes and any underlying stories of the data, carefully interpreted any direct and indirect meanings and thoroughly analysed all original data to identify alternative and new interpretations to address the limitations of coding.

The above process aimed to ensure all themes were meaning‐based, reflective with suitable values with the data, and involved analytical engagement of the existing knowledge of researchers. Third, all themes and supporting claims were then generated and refined by following an iterative process, where concepts were constantly examined, discussed, and revisited. Additional themes were created by examining the connections between key themes and subthemes across the entire dataset to ensure any potential underlying themes would be captured. Fourth, throughout the entire data analysis process, the research team frequently checked, discussed, and refined codes and themes to ensure they included the full range of opinions. Any discrepancies were resolved via discussions until achieving consensus. The research team involved researchers with advanced knowledge in OHS, HP, and OH; therefore, they positioned themselves as stakeholders with required combined knowledge to analyse the data and interpretations of the data were also shaped by researchers' values and reflections.

Also, the research team aimed to ensure a rigorous data analysis process to reveal the real voice of the sample professionals and employers on a factual basis and therefore, researchers' own perceptions were carefully discussed within the research team to avoid any likely misinterpretation of the data. The combined knowledge of the research team was particularly helpful to offer more meaningful insights into the Australian context of integrated approaches (e.g., where to be improved, what misconception of OHS or WHP is, divergence in academic disciplines). Further, the data the participants provided were of great detail and depth, achieving thematic saturation (i.e., no new information was forthcoming) during data collection and analysis.

## 3 Results

Through reflective thematic analysis, we develop four overarching themes based on the data and our careful reflections in the following subsections: (1) knowledge and attitude gaps discouraging integration; (2) multiple factors of limited integration; (3) current trends and examples of integration; and (4) employer support is always important for integration.

### 3.1 Knowledge and Attitude Gaps Discouraging Integration

OHS professionals with qualifications in public health or similar, but not health promotion, expressed basic WHP knowledge (e.g., multiple determinants of health), without being able to describe deeper and core WHP content (e.g., health behaviour change theories or program development and evaluation). However, OHS professionals without qualifications or learning or practical experiences in health promotion or related disciplines (e.g., public health, health science) expressed complete unawareness and uncertainty about WHP. The specific group of participants also demonstrated difficulties in understanding the definitions of ‘health’ and ‘wellbeing’ and were unable to describe any WHP specific strategies:

We do not look at health side, although there is a health in the middle of OHS (P3).

We're not really trained very well in the health side of things at all (P13).

OHS professionals acknowledged that their duties were safety‐focused and that the lack of health‐related knowledge in OHS professionals was very common in the industry, although the professional title ‘OHS’ includes the word ‘health’. Several OHS professionals also mentioned that in some cases their roles as safety advisors did not compulsorily require university‐level qualifications that could have equipped them with advanced knowledge and skills. Similarly, HP professionals had difficulties in understanding and describing OHS‐related terminology, such as risk management, occupational hygiene, hazard identification and control:

We only think about workplace environmental changes when it comes to very obvious exposures, such as air pollution (P22).

Participants expressed that in Australia, there exist very few professionals with both OHS and WHP knowledge:

The industry at the moment, it's really hard to recruit people that have cross section of experience (P20).

During the focus group discussions, some OHS professionals expressed the misconception that WHP was an approach to enforce individual lifestyle changes and only about building individual resilience. Some OHS professionals with health‐related knowledge contested the perceptions above, while others expressed an interest in studying health‐related courses. In addition, OHS and HP professionals frequently demonstrated differing work styles and interests. Some OHS professionals were found to ‘have no interest in wellbeing’ (P3) and some WHP professionals generally had ‘no interest in safety’ (P6).

Moreover, OHS professionals were labelled by others as ‘quite technician‐based, authoritative, not very consultative’ (P35), adopting ‘top‐down communication’ (P33) styles even though ‘there's consultation in the OHS Act’ (P33). In contrast, HP professionals appeared to others as ones who ‘get a lot better softer skills’ (P14), ‘be more inclusive’ (P11), and ‘try to bring people on the journey, they needed to influence and educate to help people’ (P45). Also, safety leadership was found to ‘be quite different with health and wellbeing leadership’ (P40).

### 3.2 Multiple Factors of Limited Integration

Our participants reported inadequate implementation of integrated approaches in Australia. They expressed the opinion that workplaces usually have regular OHS professionals with a substantial safety focus but have very occasional interactions with HP or OH professionals. The limited integration occurs mainly due to the scarce WHP implementation, with several reasons outlined in the following subthemes.

#### 3.2.1 Where Is WHP—Presence and Benefits Have Been Overlooked

One reason of limited integration is related to inadequate WHP focus within current policies. OHS and WHP sit in different government agencies in Australia and are not covered under a common legislative framework:

Even our data driven by injury, something that has occurred, rather than the absence of injury and illness, the disease outcomes and all those elements are actually reported in the health department (P36).

Legislatively, we do not have a WHP place… how legislation supports health and wellbeing programs, it's not in the legislation (P13).

The legislation is all about organisational fixing the environment… no legislation to talk about minimum requirements to use WHP (P2).

The risk management framework doesn't necessarily lend itself well to health promotion (P7).

Moreover, some participants stated that WHP normally faces challenges regarding the uncertainty of realising its benefits and the ambiguity in the duration necessary to achieve a satisfactory return on investment. First, from the perspectives of OHS professionals and employers, WHP benefits are usually invisible and occur following a long‐term period:

The immediate effects are not always felt… they're a long term… organisations don't see return on investment (P1).

Some workers don't want to come unless they know it's going to help them right now, but they don't think about the long term (P20).

If you are unsafe, you don't follow the rules applied in your organisation, you could lose your job but being unhealthy don't necessarily (P22).

#### 3.2.2 WHP Evaluation Is Very Challenging

Compared to relatively mature OHS systems in Australia, WHP evaluation is more challenging. It could be a reasonably straightforward process to collect OHS‐related data for subsequent evaluation, such as identifying information from chemical datasheets in the laboratories and then tracking the use and disposal of chemical products within the institution. However, the absent or poor quality WHP evaluations render their effectiveness unknown, making WHP implementation and scale‐up more challenging:

Get data on the number of incidents that have occurred… easy… with the health promotion side, it becomes a bit more complicated… getting data about how much it can be impacted by the workplace and the work environment somebody's working for and how much is to do outside of work… difficult (P32).

Participants shared several reasons that could explain why WHP participation and evaluation are problematic. First, regarding privacy concerns, some workers might hold the misperception that WHP is an unreasonable disruption in their personal life, leading to a low interest in evaluation, even when the company assures them that no identifying information will be collected for evaluation: ‘people are quite reluctant to share health information with their employer’ (P11). Second, the belief of ‘working hard’ is relatively common across workers, with a higher salary being more appealing than long‐term health. This means this belief likely supports the practice that workers work more to earn more money rather than spend time participating in WHP (e.g., more time to look after their own health). Third, WHP quality (e.g., language, content, delivery method, access) usually causes concerns, such as whether participation in WHP will have any implications for worker employability: ‘if you start looking at things like BMI and start excluding workers for tasks because of their BMI, that's massive… got to be careful with how we're communicating about this (WHP)… we're not adding to stigma… we are supporting workers to remain in the workforce, because that's important for their health’ (P18). Fourth, WHP evaluation might be costly: ‘in most cases, the follow‐up WHP evaluations might require support from external companies’ (P19).

#### 3.2.3 Diverse OHS Professionals' Views About WHP

In Australian workplaces, OHS professionals play a crucial role in enhancing health and safety, with HP or OH usually absent or inadequate. Among all participants, one OHS professional firmly did not support WHP implementation and believed that achieving OHS through changing the work environments was the only effective option and full responsibility of employers. The remaining participants supported OHS‐WHP integration:

That health promotion is a very important component, it's like a primary prevention strategy, you can't really have one (OHS) without the other (WHP), because you still need to have a certain level of primary prevention in order for these organisational level interventions to work. They are different but they are both needed (P25).

Additionally, participants offered explanations as to why some OHS professionals present as negative towards WHP. The predominate reason is that WHP, as an individual‐level based intervention (e.g., Employee Assistance Programs—EAP, or counselling sessions), has been traditionally recommended by employers as an ‘excuse’ to shift the responsibility back to workers instead of fixing systemic workplace problems (i.e., poor job design, excessive workload). Therefore, for the purpose of protecting workers, OHS is viewed as relatively more effective way to address actual problems, with employers taking full responsibility for the design, safety, etc. of work systems. However, overly emphasising the responsibility of employers might mistakenly weaken the real value of WHP, especially when considering the limited health‐related knowledge in OHS professionals, as presented in Section 3.1 above.

I think health and wellbeing promotion has been demonised… I think OHS professionals see workplaces rely on health promotion at a primary level, because it's easy to put it back on the worker, because employers, the easy fix is to say, you (workers) fix yourself, you (workers) make sure you eat well… (P27).

The employer must create the environment that allows a person to thrive, that you create a mentally and physically healthy workplace, that it is not up to the worker, it is up to the employer and so many OHS professionals think, my job is to make sure the employer knows, forget about that health and wellbeing, so WHP becomes demonised (P40).

#### 3.2.4 How Much We Should Be Responsible for Worker Health?

From the perspective of employers and OHS professionals, another reason for low integration is the uncertainty about, and unwillingness for, managing worker health via WHP. More specifically, some employers believed that ‘health is so complex’ (P43); given that many factors contribute to worker health, these might be work‐related and non‐work‐related, each influencing another. For example:

When you're talking about safety, usually something tangible. If we go down that path (worker health), where is that going to lead? How you manage cardiovascular diseases when it's so complex and many contributing agents (P29).

Moreover, some employers did not believe they should consider worker health because this was about:

Individuals seeking medical help, not work‐related (P32).

Similarly, regarding the management of psychosocial hazards with a mental health focus, participants stated it remains unclear how to draw an entirely clear line between worker and employer responsibilities, with workplaces having a high level of uncertainty on this topic:

People with chronic conditions might be driving a long distance, as part of job, in order to make critical work‐related decisions, difficult to set clear lines between potential work‐related and non‐work‐related risks (P35).

The likely situation to increase worker awareness of WHP importance and worker participation is the scenario that workers' health problems have become serious:

They (workers) would proactively use WHP because their lifestyles are too obvious that can badly affect work (P28).

#### 3.2.5 WHP Issues: Low Quality and Poor Implementation

Participants expressed that traditional WHP activities (e.g., providing health information) may not be most effective for optimised worker engagement, because workers can easily find relevant health information on their own. Instead, contemporary effective WHP should target work‐related factors (e.g., poor job design, bullying in the workplace) throughout the whole employment cycle (e.g., from onboarding to retirement). Good WHP requires support from all management levels in a systematic and consistent approach (e.g., all managers need to consistently encourage WHP, supported by internal and external policies).

Workers should be able to see good value in WHP and relevance to them before participating:

(WHP) that aren't so much part of the management system itself, but they're more of an employee type of benefit, but these great things need to link job design (P9).

Further, HP or OH professionals might not have adequate chances to utilise their skills and knowledge, due to the constraints of funding, job clarity, and competing interests. Our participants expressed the opinion that EAP is one of the common WHP activities in which they normally participate, while conceptually, WHP initiatives are typically more proactive, preventive, and population‐focused. Participants expressed that EAP could provide individual or group services (e.g., regular company‐wide emails listing all services, when workers seeking assistance) with various topics (e.g., work‐life balance, mental health, skin check).

Also, the quality assurance of services provided by HP or OH professionals could be crucial, because in some cases, they lack the knowledge of understanding work‐related factors to provide useful services:

If they (HP or OH professionals) don't understand our workers and the issues that they are experiencing, they may not actually be giving the right advice. It really does come down to the company and how well they've selected WHP provider… our company is growing quite rapidly, many changes… make sure that our EAP service grows with us, so that our staff are getting exactly what they need (P14).

Participants also noted that there might be some workers in need of adequate mental preparation (e.g., encouragement, confidence) to receive WHP due to personal beliefs or traumatic experiences. Those workers receiving inappropriate WHP might cause negative impacts:

Workers did their best to swallow pride, wanted to use EAP, not receiving the right advice, you will start losing people and they didn't want to use it anymore (P17).

Moreover, workplaces struggling with OHS compliance, especially the ones of small and medium size, are less likely to actively engage with WHP; whereas, interestingly, WHP might provide another path to enhance OHS:

Our business managers have indirectly showed care for WHP in our OHS systems, because they frequently seek clarification about OHS audits, they visit the workplace to support staff and address their concerns, sometimes, health issues, like extra financial support for diseases (P19).

### 3.3 Current Trends and Examples of Integration

There might be various reasons that workplaces start considering integration, such as improving worker retention and productivity. Of note, the COVID‐19 pandemic, for example, was viewed by some participants as a crucial factor that motivated integration and emphasised the importance of mental health, reinforced by Codes of Practice in managing psychosocial hazards across several Australian jurisdictions.

Some participants reported several unsuccessful integration examples, attributed to the limited collaboration between different departments within the same company. For example, in one mining company, there was limited cooperation between safety departments and health and wellbeing departments. Similarly, participants reported that: ‘Human Resources, EAP and health and wellbeing, are currently very siloing… they have to work together’ (P13), ‘these siloing not working very well, who's going to take the ownership for integration?’ (P15).

Albeit with some reported weaknesses of traditional WHP as mentioned above (e.g., simply providing health information), this ‘minimum’ service level might still be important for some work contexts. For example, mining industries require basic WHP materials because workers live in environments that are ‘very remote’ (P33), ‘away from their families’ (P2), and they might not implement work‐life balance strategies because: ‘this basic level health promotion did not occur in their organisations’ (P6).

Some successful integration examples usually occur in large‐sized workplaces, which could integrate services better because of dedicated time and resources that small‐sized workplaces could not typically afford. Nonetheless, occasionally, culture and values might also be important to support integration in small‐sized workplaces:

If they've got strong management, a really good culture, morale, and value, the workforce is more likely to get involved in integration (P8).

One company… everything is about how well you are, how you're going… they believe in the fact that if their workforce is well, then they will have a great business… they have a very high retention rate and a low injury rate (P24).

The successful ones are where they actually listen to workers (P35).

### 3.4 Employer Support Is Always Important for Integration

Some OHS professionals reported that, in some cases, employers heavily rely on them to lead both OHS and WHP initiatives. Interestingly, some participants stated that managers at different organisational levels might have different views about integration. For example, middle‐level managers tend to have more knowledge and demonstrate more support for integration, with top‐level managers considering broader impacts of integration. Also, organisational size might influence the attitudes and knowledge of employers.

I think if leaderships don't have the capability to understand how the two can be integrated, they will rely heavily on their WHS people or their people and culture people to tell them what to do. If those people are not good at integration, that's going to be a problem. The leaders need to have better capability and understanding of how integration will help. I think the people who are trying to influence them, need to come together and make sure that they've got an integrated story (P32).

If we're talking about small businesses or micro, they're generally family businesses… produces their own values. When you get into the medium‐sized businesses, it might be that they don't have an opportunity to get management training, because sometimes very output focused, but in large businesses, you have the opportunity for your managers to do professional development… they want their big companies to be world‐leading in everything, including safety and health (P29).

If it's (WHP) at no cost to the employer, you (HP) might have a chance. When they have to pay money to do it, they do not understand worker health and wellbeing, they see people perhaps being taken away from work, there's a danger that productivity could go down, employers will wait for you (HP) to fail (P3).

## 4 Discussion

The recent cross‐sectional study found limited integration implementation in Australian workplaces [19], this focus group and interview study reveals important insights into the implementation context of integrated approaches in Australia. It highlights important reasons for limited integration, including policy emphasis, knowledge and practice gaps in professions, and WHP implementation‐ and evaluation‐related concerns. While consistent with the findings from a recent study [18], organisational size was an important factor influencing the implementation context of integrated approaches in Australia, several other key considerations are further discussed below.

### 4.1 Worker Health and Capacity Building

Given that OHS policies heavily focus on safety [28], to support integration, it is important to start establishing a cultural shift to ensure ‘health’ becomes an increasingly active element in OHS initiatives and management systems. Safety should be considered as a function of managing risks to ensure worker health, instead of the goal and destination [29]. Future OHS policy development should include health‐related elements and processes, such as the provision and quality assurance of WHP services and lists with minimum EAP requirements, which are not visibly emphasised in current policy frameworks. For example, these are particularly required when addressing psychosocial hazards in the workplaces [28, 30, 31]. In this case, for intervention implementers, adequate competencies of properly understanding the knowledge of health and wellbeing and well‐trained communication skills are important to support worker mental health (e.g., stress, fatigue) [28, 30, 31]. However, the above can raise questions about whether WHP should be considered a legal or moral responsibility. As, traditionally, OHS professionals are instructed to not focus on individual workers [32, 33], the fundamental notion of WHP is that of providing healthier choices for individuals and communities through voluntary participation [34]. Such notion might not be in the interest of any stakeholder to be regulated through policies; instead, the intention of future policy development should focus on building capacity to support WHP. This process requires extensive and meaningful consultations between policy makers, regulators, OHS professionals, health professionals, employers, and employees, an approach similar to the one supported by Halliday et al. [35].

On the other hand, there is a potential concern that OHS professionals without health‐related knowledge might unintentionally contribute to negative impacts when planning, implementing, and evaluating WHP (e.g., for managing psychosocial hazards). Such impacts can include low participation and satisfaction in WHP, misleading health‐related information, and unnecessary fears regarding health and wellbeing management (e.g., inappropriate language about health behaviour change) [34, 36, 37].

Indeed, little is known about how OHS professionals manage psychosocial hazards in Australia. However, based on our sample participants' perspectives, the basic WHP content, such as encouragement instead of enforcement, and health literacy and efficacy were not always covered in their existing OHS education and training sessions. Hence, it is likely that OHS professionals might pass the information of WHP‐related uncertainty or misconceptions to other stakeholders, which can render OHS‐WHP integration challenging.

Similar to our findings, Svendsen et al. observed that OHS professionals in Denmark had limited awareness in managing musculoskeletal disorders [38]. In another study by Moore et al. focusing on industry skills in OHS graduates [39], health‐related knowledge and skills were completely absent. Moreover, Inoue et al. noted that OHS auditors in Japan normally lacked confidence and skills in assessing occupational health issues (e.g., skin diseases, cancer), leading to barriers to properly assessing and controlling occupational health risks and warranting more educational opportunities [40].

Nevertheless, in an earlier review, Wang et al. suggested that OHS research trends have shifted from a main focus on musculoskeletal disorders to considering broader disease prevention and management through a public health and preventive health approach [41]. Therefore, providing and enhancing health‐related training for OHS professionals appears to be an urgent intervention effort, especially in face of managing psychosocial hazards and other types of hazards that are not physically visible (e.g., noise and radiation). These knowledge gaps call for curriculum innovation efforts at the tertiary education level so that graduates become better prepared for a role that integrates WHP and OHS.

### 4.2 Systems Thinking in Contemporary WHP

Our study suggests that contemporary WHP requires innovative thinking and design to ensure WHP closely links to workplace needs by adopting a systems thinking approach [42]. More specifically, WHP should transcend its traditional scope and act on work‐related factors, such as job design throughout the whole employment cycle. A recent systematic review by Javanmardi et al. noted that most WHP interventions were still focused on individual behavioural changes, with scant cases of emphasis on environmental changes due to barriers from employers and workplace policies [43].

Guided by the KTA framework [22], we synthesised and analysed the data from our sample participants (e.g., in knowledge creation phase: from knowledge synthesis to knowledge tools/products) and reflected on our own values and practices to suggest several directions for contemporary WHP with some examples in Table 2.

**TABLE 2:** Directions for contemporary WHP with some examples.

Suggestions	Examples
OHS and health professionals work closely with employers in influencing work designs	Establishment of specialised departments for all required health and safety professionals
	Internal communication systems with a holistic view of worker health and safety
The quality of, and satisfaction from, WHP is timely monitored and improved	Internal policies and procedures to receive and address feedback about WHP
	Training of WHP implementation staff for quality assurance, e.g., onsite medical doctors with proper knowledge and understanding of workplace
Workers are supported via WHP in an ongoing, consistent, and systematic manner throughout the entire employment cycle	Education of WHP notions (e.g., health equity) delivered in all workers and managers
	Job redesign with the consideration of worker health and wellbeing at all stages (e.g., daily work, work recognition, retirement)
Employers formally include OHS and health professionals in the broader management and decision‐making processes	Health and safety professionals are involved in managers' meetings, support workers to attend job‐related meetings

### 4.3 Integration as a Linkage Process

One important barrier to integration could be the absence of the notion that ‘integration could be a linkage process’, which, based on systems thinking, can underpin the coordination of separate WHP and OHS initiatives through more collaboration between departments. For instance, social services employers can focus primarily on the design of work and the reduction of risks through effective controls. Workers, instead of visiting an aggressive client, can choose to talk with the client over the phone to minimise potential psychological and physical harm, while WHP should have the role of supporting workers to develop coping skills. However, such a notion was not evident in our sample. One possible practical direction is that more meaningful efforts may be required to improve the visibility of WHP usefulness, a finding also revealed by Provan et al. [9].

Furthermore, although systems thinking seems to be an important approach, overly emphasising organisational factors and underappreciating the role of individuals across the organisation may impair the feasibility of integration and could slow the integration process. Especially in small and medium‐sized enterprises, which may not have readily available sources to pursue integration, it is important to focus first on small workable areas instead of changing entire systems and start with small steps such as WHP educational opportunities, identification of work‐related issues of higher priority [1], and evaluation and revision of existing EAP programs before introducing additional WHP elements.

Given that large‐sized enterprises have relatively more potential to achieve good OHS‐WHP integration, a formative evaluation in the integration planning process in small and medium‐sized enterprises can prove to be insightful and useful to generate more empirical research evidence [44]. The KTA Framework [22], for example, can serve as a promising theoretical and practical tool to translate the above knowledge (knowledge creation) into action (knowledge application) in the entire integration planning, implementation, evaluation, and revision processes.

In addition, as part of reflective thematic analysis, we employed the KTA Framework as a tool to help collect and analyse the data, given that our research team involved full expertise of OHS, HP, and OH. This allowed us to stay in a ‘neutral’ position to read the data and understand any potential stories behind the data; therefore, our data analysis process, guided by the KTA Framework, could be considered a transition from knowledge synthesis to knowledge tools/products.

Importantly, when transforming from knowledge ‘creation’ to ‘action’ in the context of integrated approaches, we note several important knowledge gaps. Predominantly, urgent intervention efforts should first be about providing more educational opportunities for employers and OHS professionals to shift the way to consistently and holistically consider worker health and wellbeing. The knowledge and practice of these stakeholders contribute to the level of implementation and effectiveness of integrated approaches, consistent with the notion of ‘knowledge review and selection’ in the KTA Framework.

### 4.4 Study Limitations and Implications for Research, Policy and Practice

Extensive educational opportunities emerge from our research for employers, OHS, HP and OH professionals, with the aim of broadening thinking and advancing skills at multiple levels. However, this study is not free of limitations. First, the participants in our sample were largely OHS professionals. Therefore, the findings may not capture adequate opinions from the professionals of HP, OH, or those with combined knowledge who may offer more balanced and comprehensive perspectives (e.g., how employers support workers with health conditions).

Second, our study did not include an adequate number of government bodies and employers who could have provided more in‐depth information. Third, given the diversity of consultancy‐related experiences in the sample OHS and HP professionals, our findings lack information related to specific occupational settings. However, as the research team encouraged all participants to recall their interactions with any other relevant professionals, the findings could be considered fundamental in understanding diverse contexts of integrated approaches in Australia and provide a good starting point for future research.

The implications of our study for research, policy, and practice are listed below. First, given our sample mostly comprised OHS professionals, future research should focus on understanding the perspectives from government bodies (e.g., regulators, worker compensation) and the opinions from more employers and OH and HP professionals. This is crucial as during the discussions the majority of OHS professionals reported no‐to‐little interactions with HP and OH professionals, which could possibly suggest the limited influence of HP and OH professionals in workplace settings, or could reflect that our sample did not include many OHS professionals with such experience. Second, as employers in this study, particularly from small and medium‐sized enterprises, generally expressed a low intention to engage with WHP, future research is required to understand: how employers decide about and manage integration in consultation with OHS, HP, and OH professionals; and how to advocate WHP in small and medium‐sized enterprises. Third, despite the reality of limited resources, small and medium‐sized enterprises may have the potential for good integration in a small‐scale and in‐depth approach, if supported with effective leadership and positive workplace culture. This provides many opportunities for researchers to conduct exploratory and intervention studies, building connections between research, practice, and policy. Fourth, WHP should expand from reactive initiatives (e.g., rehabilitation and return to work services) to broader and more proactive services and closely target work‐related factors in the entire employment cycle. To achieve this, organisational and individual values and knowledge must be seen as crucial factors for successful integration, which could be a linkage process in the service delivery spectrum and enhanced at multiple levels. Future policy development can start with WHP recommendations as a pilot, with the reference of existing effective health and wellbeing guidelines.

## 5 Conclusions

This qualitative study provides important insights into the context of integrated approaches in Australia. The integration barriers could be related to knowledge gaps, particularly regarding OHS professionals' limited understanding of WHP and HP professionals' difficulties with OHS terminology. Reasons for inadequate integration implementation include scarce WHP implementation, invisible WHP benefits, poor WHP evaluation, differing views of OHS professionals about WHP, debate about worker health, and WHP quality and implementation issues. The trends of integration are often motivated by factors like COVID‐19 and the integration occurs more successfully in large organisations, though often hindered by departmental siloing. Roles, attitudes, and knowledge of employers, with varying support for integration across management levels and organisational sizes, are important factors impacting integration. Overall, limited implementation of integrated OHS‐WHP approaches in Australia stems from lack of policy emphases, professional knowledge gaps, and WHP implementation and evaluation concerns.

## Author Contributions

**Yanming Lu:** conceptualization, methodology, writing – original draft, writing – review and editing. **Nektarios Karanikas:** conceptualization, methodology, writing – review and editing. **Julie‐Anne Carroll:** conceptualization, methodology, writing – review and editing.

## Funding

The authors have not received any funding to support this research.

## Disclosure

The authors have nothing to report.

## Ethics Statement

The study was approved by Queensland University of Technology Human Research Ethics Committee (Ethics Number: 8350).

## Conflicts of Interest

The authors declare no conflicts of interest.

## Data Availability

The data that support the findings of this study are available from the corresponding author upon reasonable request.

## Appendix A

Discussion Guide

I would like to start by thanking you very much for making the time to take part in this discussion today. The participant information sheet has been sent to you before this session. I would like to record this session. During the discussion, your identity will unlikely remain anonymous. However, please rest assured that all information extracted and reported from our conversation will be anonymous. Every effort will be made to ensure that the data you provide during this session cannot be traced back to you in reports, publications, and other forms of presentation. Please do not share with others any private and confidential information and do not disclose to third parties after the session any information that will be discussed today. I would like to also stress that your participation in this focus group or interview is completely voluntary, and you are able to leave this discussion at any time without any harms.

Definition of workplace integrated approaches

The main purpose of this discussion is to talk about your experiences and perceptions of workplace integrated approaches. I would like to do some warm‐up activities first. I will ask some questions; you could add a few sentences after them.

- *Occupational health and safety (OHS) is about …*
- *Workplace health promotion (WHP) is about …*
- *As a business manager/work health and safety professional/health promotion professional/occupational health professional, I think integrated OHS and WHP would be about …*

Sharing of the main findings of a previous study

Thank you. I will ask some questions regarding workplace integrated approaches. Before this, I would like to present the results of the survey I recently completed in a broad range of Australian workers.

Main discussion

1. How do you feel about the extent to which integrated approaches are currently implemented in Australia?
2. Has your workplace implemented any integrated approaches—why/why not?
3. Why do you think OHS interventions, WHP interventions, or integrated approaches are implemented separately or together in Australia?
4. As a business manager/work health and safety professional/health promotion professional/occupational health professional, do you think employees are willing to participate in integrated approaches?
5. What types of integrated approaches do you think employees need—why?
6. Has your workplace encouraged workers to participate in any integrated approaches? If so, could you talk a bit more?
7. Have you known some successful or unsuccessful integrated approaches in Australia?
8. Do you have any concerns about integrated approaches? (Prompts: What are they? Why?)

Thank you very much for your discussion. We are about to finish this session; is there anything else you would like to add?
